# Association of Hospital Admission Risk Profile Score with Mortality in Hospitalized Older Adults

**DOI:** 10.1093/geroni/igx007

**Published:** 2017-08-02

**Authors:** Stephen K Liu, Marshall Ward, Justin Montgomery, John N Mecchella, Rebecca Masutani, Stephen J Bartels, John A Batsis

**Affiliations:** 1 Section of General Internal Medicine, Dartmouth-Hitchcock Medical Center, Lebanon, New Hampshire.; 2 Geisel School of Medicine at Dartmouth, Hanover, New Hampshire.; 3 Section of Rheumatology, Dartmouth-Hitchcock Medical Center, Lebanon, New Hampshire.; 4 Department of Psychiatry, Dartmouth-Hitchcock, Lebanon, New Hampshire.; 5 Centers for Health and Aging, Dartmouth College, Lebanon, New Hampshire.; 6 The Dartmouth Institute for Health Policy & Clinical Practice, Lebanon, New Hampshire.

**Keywords:** Acute/short-term care, Decision making, Hospital

## Abstract

**Objectives:**

To evaluate the association of the Hospital Admission Risk Profile (HARP) score with mortality after discharge in a population of hospitalized older adults.

**Design:**

Retrospective cohort study.

**Participants:**

Hospitalized patients aged 70 years or older.

**Measurements:**

Patient age at the time of admission, modified Folstein Mini-Mental Status Exam score, and self-reported instrumental activities of daily living 2 weeks prior to admission were used to calculate a HARP score. The primary outcome assessed was overall mortality up to 365 days after hospital discharge. Cox proportional hazard analyses evaluated the association between HARP score and mortality adjusting for age, sex, and comorbidities associated with increased mortality.

**Results:**

Of the 474 patients, 165 (34.8%) had a low HARP score, 177 (37.4%) had an intermediate, and 132 (27.8%) had a high score. HARP score was not associated with differences in 30-day readmission rates. High HARP score patients had higher mortality when compared to patients with low HARP scores at all time frames (30 days: 12.9% vs 1.8%, *p* < .05; 90 days: 19.7% vs 4.8%, *p* < .05; 365 days: 34.8% vs 16.9%, *p* < .05). In fully adjusted Cox proportional models, patients with high HARP scores had a 3.5 times higher odds of mortality when compared to low HARP score patients.

**Conclusion:**

The HARP score is a simple and easy to use instrument that identifies patients at increased risk for mortality after hospital discharge. Early identification of patients at increased risk for mortality has the potential to help guide treatment decisions following hospital discharge and provides additional information to providers and patients for shared decision making and may help in clarifying and achieving patient and family goals of care.

**Translational Significance**

This study found that patients admitted with a high HARP score have a higher mortality risk after discharge when compared to those classified as having a low or intermediate HARP score. These results suggest that the HARP score can help providers identify patients at risk for increased mortality after discharge and facilitate discussions of post-discharge prognosis and care planning in order to better align treatment plans with patients’ values and goals.

## Background and Objectives

The acute hospitalization of older adults can represent a major health transition point which provides an opportunity to identify issues and values which matter most to a patient and clarify goals of care following hospital discharge. A large majority of hospitalized older patients are interested in discussing end-of-life treatment planning and overall prognosis after discharge with their physicians (Miles, Koepp, & Weber, 1996; Reilly et al., 1994). Aligning any proposed health care interventions after hospital discharge with those self-identified goals of care and values can help achieve the best possible outcomes for patients. A simple to use prognostic index that would estimate mortality following hospital discharge would be useful to inform this conversation with patients and family members.

Published mortality predictors and risk scores are often cumbersome to use or require the use of administrative or laboratory data which are not typically used or readily available in routine clinical care (Pilotto et al., 2012; Teno et al., 2000; van Walraven, 2014; Walter et al., 2001). Some of the previously published studies about mortality scoring systems have evaluated patients with single diagnoses such as chronic obstructive pulmonary disease, dialysis, or congestive heart failure (Fuso et al., 1995; O’Connor et al., 2008; Ranieri et al., 2008; Thamer et al., 2015), which may not be applicable in geriatric patients with multiple co-occurring medical conditions. Others, including the Charlson Comorbidity Score and the Acute Physiology and Chronic Health Evaluation (APACHE) Score, use patient information that is not typically used or obtained in clinical care for nonintensive care unit patients (i.e., blood pH, arterial partial pressure oxygenation [PaO2], or serum albumin levels) or involve the summation of multiple patient comorbidities that may not be readily available within the medical record or would be time consuming to perform in a clinical setting (Christensen, Johansen, Christiansen, Jensen, & Lemeshow, 2011; de Gelder et al., 2016; Martinez-Velilla, Cambra-Contin, & Ibanez-Beroiz, 2014; Walter et al., 2001).

In populations of older adults, decreased functional capacity and increased care dependencies are known to be associated with increased patient mortality and rates of institutionalization (Boyd et al., 2008; Covinsky et al., 2000; Covinsky et al., 2003; Ferrucci, Guralnik, Pahor, Corti, & Havlik, 1997; Mudge, O’Rourke, & Denaro, 2010; Takata et al., 2013). Multiple factors have been associated with functional limitations including incontinence, impaired vision, malnutrition, cancer, diabetes, coronary artery disease, chronic lung disease, and low body mass index (Abicht-Swensen & Debner, 1999; Cohen-Mansfield, Marx, Lipson, & Werner, 1999; Dale, Burns, Panter, & Morris, 2001; Dubeau, Simon, & Morris, 2006; Ferrucci et al., 1997; Guccione et al., 1994). Many of these risk factors are also independently associated with increased mortality(Charlson, Pompei, Ales, & MacKenzie, 1987) and a method to identify patients at risk for functional decline may also have the ability to identify patients for increased mortality.

The Hospital Admission Risk Profile (HARP) is a clinical instrument that was designed to identify patients at risk for functional decline during a hospitalization. In the original study published in 1996 (Sager et al., 1996), a cohort of older hospitalized patients were utilized to identify variables significantly associated with declines in ADL function following discharge. Advanced patient age, limitations of instrumental activities of daily living (IADL) 2 weeks prior to admission, and impaired cognitive function measured using an abbreviated Folstein Mini-Mental Status Examination (MMSE) were significantly associated with declines in ADL function after hospitalization. By summing these variables patients were classified into low, intermediate, and high-risk categories, where each category had a higher predictive risk of functional decline (Sager et al., 1996). We hypothesized that the use of an easily administered HARP score, which predicts functional decline during a hospitalization, can additionally predict mortality following hospital discharge.

## Research Design and Methods

### Study Setting and Participants

This study was performed at Dartmouth-Hitchcock Medical Center, a rural, academic 396-bed tertiary care hospital located in Lebanon, New Hampshire. Dartmouth-Hitchcock Medical Center serves a population of 1.5 million people primarily from rural areas in New Hampshire and Vermont and has approximately 25,000 discharges annually (Dartmouth-Hitchcock Medical Center, Facts and Figures, 2015). Patients included in this study were aged ≥70 years and admitted to a single 35 bed internal medicine inpatient unit from October 1, 2013 to June 1, 2014. During this time period, eligible patients were enrolled into the study consecutively within 24 hr of admission to the inpatient unit.

The study was approved by the Committee for the Protection of Human Subjects at Dartmouth College and granted a waiver for signed informed consent. Patients and families were given an information sheet upon enrollment describing the quality improvement initiative that was implemented in the unit, the collection of selected patient data and given the option to not participate or not have their data recorded in the database.

All hospitalized patients were internal medicine and medicine subspecialty patients managed by a hospitalist service covered by both resident and attending staff physicians. Patients were excluded from the study if a complete geriatric screening (described below) was not completed upon admission due to an inability to obtain information from the patient or family members, patient transfer to another service or unit, or patient discharge prior to completion of the admission screening.

Geriatric admission screenings were implemented in the inpatient unit as a component of a larger quality improvement initiative to improve care for hospitalized older adults using a team of geriatric trained licensed nursing assistants (LNA). Enrolled patients received a geriatric screening after admission to the unit by a specially trained geriatric LNA and the LNA would calculate a HARP score based on the data obtained from the admission screening. The HARP score was then used to identify patients at increased risk for functional decline who would receive additional mobilization and activities guided by the geriatric LNAs during the hospitalization.

The geriatric screening included the patient’s age at the time of hospital admission, patient or family self-reported activities of daily living and instrumental activities of daily living (ADL and IADL) (Katz, 1983) 2 weeks prior to admission, living situation and support prior to admission (home, home with assistance, assisted living facility, nursing home/skilled nursing facility, transfer from outside hospital), cognitive screening using the Folstein MMSE (Folstein, Folstein, & McHugh, 1975), and the patient’s desired discharge disposition. The screenings were reviewed by a supervising geriatric nurse practitioner who could address any concerns identified with the admission screenings.

**Figure 1.**
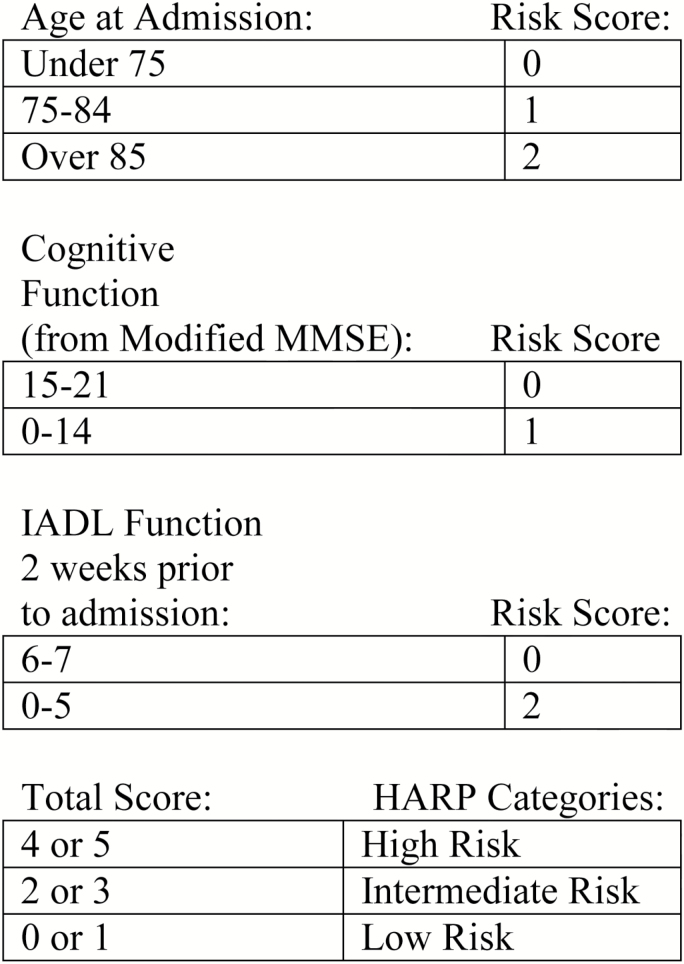
Hospital Admission Risk Profile (HARP) scoring system used to calculate HARP score based on patient age, modified Folstein Mini-mental Status Exam and Instrumental Activities of Daily Living 2 weeks prior to admission. Adapted from (Sager et al., 1996). IADL = Instrumental Activities of Daily Living; MMSE = Modified Mini-Mental Status Exam.

The three components of the HARP score include age, cognitive function, and IADL function 2 weeks prior to admission. For age, there are three age categories, under age 75, age 75–84, and ≥ 85. Patients aged 75–84 years were assigned one point and patients ≥ 85 were assigned two points. Scores for cognitive function were calculated using an abbreviated Folstein MMSE that omits the language items which included naming, repetition, three-stage command, reading, writing, and copying tasks. This resulted in a 21 item modified MMSE that assessed orientation (10 items—year, season, month, date, day, city, county, state, hospital, floor), registration (3 item identification), attention (5 item—spelling world backwards), and recall (3 item recall from item registration task). Patients with a modified MMSE score of 15–21 were assigned 0 points and patients who scored 0–14 were assigned 1 point (Sager et al., 1996). Scores for IADL function were assigned based on patient reported ability to complete an activity independently without assistance 2 weeks prior to hospital admission. The seven IADLs evaluated included managing finances, taking medications, use of the telephone, shopping, transportation, housekeeping, and food preparation. Patients who were independent in 6–7 tasks were assigned 0 points and patients who were independent in 0–5 tasks were assigned 2 points. The total HARP score was calculated by summing points in each of the three categories and patients with a total score of 4–5 points were assigned to the high risk category, scores 2–3 were assigned to intermediate, and scores of 0–1 were assigned to the low risk category (Sager et al., 1996). See Figure 1 for an overview of the scoring system used to calculate the HARP score.

The patients’ electronic medical record numbers were recorded in a secure database and used to query the Dartmouth-Hitchcock data warehouse to obtain additional patient data for the purposes of this study. Data obtained resided on secure institutional servers maintained in accordance with institutional data security standards. The recorded data represented demographic information (age, gender), clinical information (height and weight at the time of admission used to calculate body mass index (BMI), selected comorbidities), and hospitalization information (admission and discharge dates to calculate hospitalization length of stay and discharge disposition).

Hospitalization length of stay was calculated from the documented discharge and admission dates. Comorbidities were based on internal billing codes using International Classification of Disease, Ninth Edition (ICD-9) and Current Procedural Terminology (CPT®) codes as described below for inpatient and outpatient visits and dichotomized (present/absent). A patient was noted to have a specific comorbidity if they had two or more occurrences of a diagnosis code over any period of time with at least one diagnosis code within the last 24 months, or ≥1 applicable CPT® code at any time. Defined comorbidities included coronary artery disease, cancer, chronic obstructive pulmonary disease, diabetes mellitus, heart failure, renal disease, and vascular disease. Comorbidity identification and BMI data was validated via manual chart review by members of the study team.

The primary outcome assessed was overall mortality. Patients were followed for up to 365 days following hospital discharge or until the time of death whichever occurred first. Time of death was calculated from the date of hospital discharge to the date of death measured in days and was determined by using information extracted from the electronic health record. When a known date of death was obtained, it was corroborated with additional information documented in the patient’s medical record (e.g., progress notes, telephone encounter with hospice or scanned death certificate) through additional medical record review. If a date of death was not present in the medical record, the date of last known contact was obtained using the date of the most recent patient encounter recorded in the record. Encounters ranged from telephone calls, progress notes, labs, imaging studies, procedures, and scanned documents from outside providers or institutions indicating the patient was alive at the time of evaluation. Patients who died during the hospitalization were excluded from the analysis of mortality. Thirty day readmissions were evaluated and defined as any hospitalization (both inpatient and observation admissions) at our institution within 30 days of the original hospital discharge regardless of admission diagnoses or inpatient service.

Continuous data are presented as means ± *SD*, and categorical data as counts and percentages. Using the calculated HARP score on admission to the hospital, patients were classified into three cohorts—low (HARP score 0–1), intermediate (HARP score 2–3), and high (HARP score 4–5). An analysis of variance assessed differences between HARP group and continuous variables, and Cochran–Mantel–Haenszel tests for discrete variables. The primary outcome evaluated was overall mortality following hospitalization. All models excluded individuals who died during the hospitalization. Our primary predictor was HARP group (low HARP=referent). Cox proportional hazard analyses evaluated the risk of death adjusting for the following covariates: Model 1—unadjusted; Model 2—age and sex; Model 3—age, sex, diabetes, cancer, chronic obstructive pulmonary disease, heart failure, renal disease, and vascular disease. These comorbidities were selected as they are key clinical variables included within the Charlson Comorbidity Score and associated with an increased risk of mortality (Charlson et al., 1987). We separately created models using HARP as a continuous variable (HARP scores 0–5). Lastly, we ascertained the impact of each HARP component individually (age, cognitive impairment, IADL impairment) on mortality in separate models after fully adjusting for covariates. We present hazard ratios with 95% confidence intervals. All statistical tests were two-sided, and *p* values less than .05 were considered statistically significant. Analyses were performed using STATA v.12 (College Station, TX).

## Results

Of the 474 hospitalized patients who had complete data, 165 (34.8%) patients had a low HARP score, 177 (37.4%) had an intermediate HARP score, and 132 (27.8%) had a high HARP score. We excluded 118 patients who were admitted from October 1, 2013 to June 1, 2014 due to incomplete data fields or admission screenings. Mean age of the overall cohort was 80.4 ± 7.34 years, 49.6% were women, and 90.1% were admitted from home. Patients in the high HARP score group were more likely to be women (59.1%), older (85.9 ± 7.3 years), and less likely to be admitted from home (82.6%). Patients in the three HARP score groups did not differ significantly in the total number of documented comorbidities during the hospitalization with an average of 3.58 ± 1.83 comorbidities per patient in the overall cohort. No individual comorbidity was significantly different among the three groups except for a diagnosis of cancer which was more prevalent in the low and intermediate groups. The average BMI for the overall cohort was 27.0 ± 6.67 and the high HARP score group had a significantly lower BMI of 25.9 ± 6.35 (*p* = 0.028). See Table 1 for demographic details of the overall and three HARP cohorts.

**Table 1.** Baseline Demographic Characteristics of Study Patients—Overall Cohort and by HARP Score

		HARP score			
	Overall cohort	Low (0–1)	Intermediate (2–3)	High (4–5)	*p* value
Number	474	165 (34.8%)	177 (37.4%)	132 (27.8%)	
Female (%)	49.6%	43.0%	48.6%	59.1%	<.05
Age, years	80.4 ± 7.34	76.0 ± 4.7	80.2 ± 6.6	85.9 ± 7.3	<.05
Comorbidities					
CAD	187 (39.5%)	59 (35.5%)	81 (45.8%)	47 (35.6%)	.09
Cancer	144 (30.4%)	58 (34.9%)	62 (35.0%)	24 (18.2%)	<.05
COPD	136 (28.7%)	48 (28.9%)	60 (33.9%)	28 (21.1%)	.05
Diabetes mellitus	156 (32.9%)	48 (28.9%)	65 (36.7%)	43 (32.6%)	.32
Heart failure	156 (32.9%)	50 (30.1%)	66 (37.3%)	40 (30.3%)	.29
Renal disease	187 (39.5%)	58 (34.9%)	80 (45.2%)	49 (37.1%)	.13
Vascular disease	300 (63.3%)	93 (56.0%)	121 (68.4%)	86 (65.2%)	.06

*Note*: All values represented are mean ± *SD*, or counts (percent).

CAD = Coronary artery disease; COPD = Chronic obstructive pulmonary disease; HARP = Hospital Admission Risk Profile; SNF = Skilled nursing facility.

**Figure 2.**
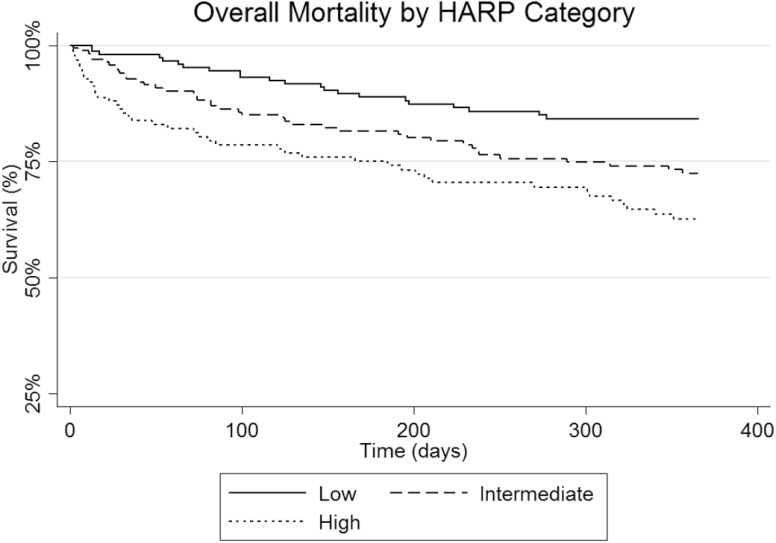
Overall mortality 365 days after hospital discharge by HARP category (low, intermediate, high). HARP = Hospital Admission Risk Profile.

**Figure 3.**
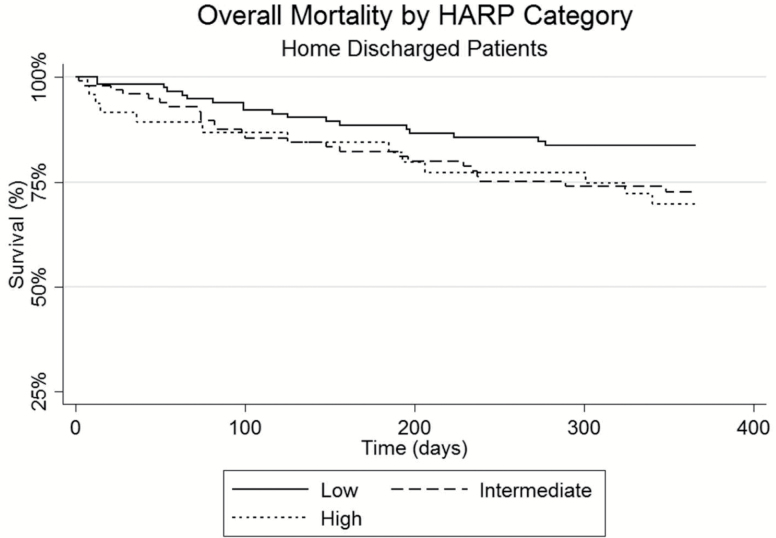
Overall mortality 365 days after hospital discharge by HARP category for patients discharged to home. HARP = Hospital Admission Risk Profile.

**Figure 4.**
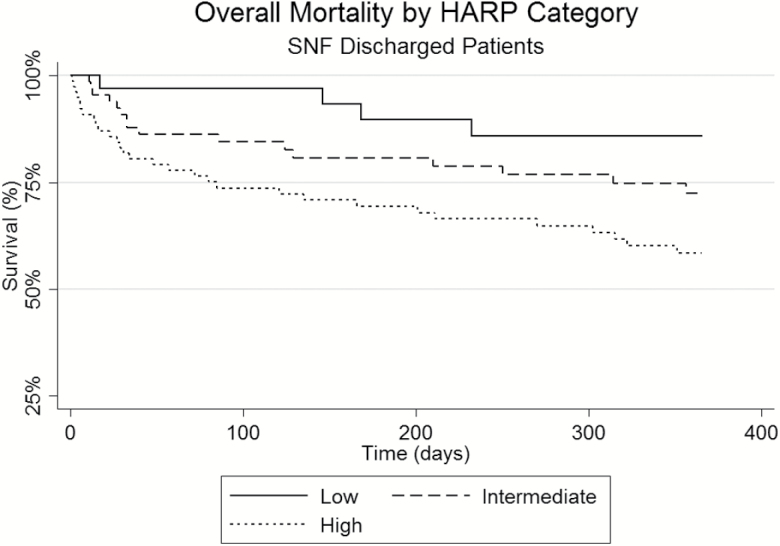
Overall mortality 365 days after hospital discharge by HARP category for patients discharged to a skilled nursing facility. HARP = Hospital Admission Risk Profile.

Table 2 details the rates of hospital length of stay, discharge disposition, 30-day readmissions, and mortality at 30, 90, and 365 days following discharge for the overall and three HARP score cohorts. Patients in the three HARP score cohorts had similar hospitalization length of stays with an overall average of 8.4 ± 22.3 days and had similar inpatient mortality rates. Patients in the high HARP score group were significantly more likely to be discharged to a skilled nursing facility when compared to the low HARP score patients (61% vs 21%; *p* < .05). Hospital readmissions were not found to be significantly different among the three HARP groups with the high HARP group having a lower readmission rate at 10.6% compared to 17.0% of the low HARP score patients (*p* = .166). Patients in the high HARP cohort had increased unadjusted mortality rates of 12.9 % at 30 days as compared to 1.8% in the low HARP group (*p* < .05) and at 365 days, 34.8% of the high HARP group were deceased compared to 16.9% of the low HARP patients (*p* < .05). See Figures 2–4 for survival plots for the three HARP score groups by overall cohort and by discharge disposition (home and skilled nursing facility) 365 days following hospital discharge.

**Table 2.** Hospitalization LOS, Inpatient Mortality, Discharge Disposition, 30-day Readmissions and Mortality in the Overall Cohort and by HARP score

		Admission HARP score			
	Overall	Low (0–1)	Intermediate (2–3)	High (4–5)	
	*n* = 474	*n* = 165	*n* = 177	*n* = 132	*p* value
LOS (days)	8.4 ± 22.3	10.3 ± 32.9	7.2 ± 10.0	7.58 ± 16.7	.419
Discharge disposition					
Home	281 (59.3%)	128 (77.6%)	104 (58.8%)	49 (37.1%)	<.05
SNF/Rehab facility	183 (38.6%)	35 (21.2%)	68 (38.4%)	80 (60.6%)	<.05
Deceased	10 (2.1%)	2 (1.2%)	5 (2.8%)	3 (2.2%)	*
Readmitted within 30 days	74 (15.6%)	28(17.0%)	32 (18.1%)	14 (10.6%)	.166
30-day mortality	31 (6.5%)	3 (1.8%)	11 (6.2%)	17 (12.9%)	<.05
90-day mortality	57 (12%)	8 (4.8%)	23 (13.0%)	26 (19.7%)	<.05
365-day mortality	120 (25.3%)	28 (16.9%)	46 (26.0%)	46 (34.8%)	<.05

*Note*: HARP = Hospital Admission Risk Profile; LOS = Length of stay for hospitalization; SNF = Skilled nursing facility.

*Sample size too small in cells to test differences.

Table 3 displays the multivariate Cox proportional hazard models analyzing the risk of death following hospitalization by admission HARP score (low, intermediate, and high). In the fully adjusted model that included age, sex and comorbidities (Model 3), high HARP score patients had a 3.5 times higher risk of death compared to the low HARP patients.

**Table 3.** Multivariate Cox Proportional Hazard Model Analysis of the Association of Mortality at 365 days After Discharge by Admission HARP score

	Model 1	Model 2	Model 3
Low HARP	Ref	Ref	Ref
Intermediate HARP	1.87 (1.11–3.15)	2.04 (1.20–3.48)	1.82 (1.06–3.14)
High HARP	2.80 (1.67–4.67)	3.45 (1.91–6.23)	3.46 (1.89–6.31)
Age	---	0.99 (0.96–1.01)	0.99 (0.96–1.02)
Sex	---	0.74 (0.50–1.10)	0.86 (0.58–1.29)
Diabetes			1.23 (0.81–1.87)
Coronary artery disease			1.06 (0.67–1.67)
Cancer			2.24 (1.48–3.37)
COPD			1.13 (0.73–1.75)
Heart failure			1.11 (0.71–1.76)
Renal disease			0.86 (0.57–1.29)
Vascular disease			1.40 (0.89–2.20)

*Note*: All values listed are represented as hazard ratios with 95% confidence intervals. Model 1—Unadjusted. Model 2—Adjusted for age, sex. Model 3—Adjusted for age, sex, diabetes mellitus, coronary artery disease, cancer, chronic obstructive pulmonary disease (COPD), heart failure, renal disease, and vascular disease.

COPD = Chronic obstructive pulmonary disease; HARP = Hospital Admission Risk Profile.

When the HARP scores were analyzed as a continuous variable, the results were similar as when the HARP scores were analyzed as three discrete categories. We also evaluated each of the three HARP components (age, cognitive function, and IADL function) independently. After adjusting for age, sex, and comorbidities associated with an increased risk of mortality, age (HR 1.36 [0.56–3.31]) and impairment in IADLs (HR 1.59 [0.99–2.53]) were not found to be independently associated with an increased mortality risk. Impairment in cognition (HR 4.50 [2.05–9.87]) was the only HARP variable found to be independently associated with an increased mortality risk after discharge.

## Discussion and Implications

Patients with high HARP scores on admission were found to have a 3.5 times greater risk of mortality 1 year after hospital discharge when compared to low HARP score patients. While the HARP score has previously been reported to be associated with loss of ADL function after discharge and facility placement after discharge (Liu et al., 2016; Sager et al., 1996), to our knowledge, this is the first study to evaluate mortality after discharge using this simple and practical tool.

After adjustment for age and comorbidities, we found that impairment in cognition was independently associated with an increased mortality after discharge, while age and impairment in IADL function were not. This was a significant finding as our initial hypothesis for this study was based on the well-known association between functional decline and increased mortality risk. This finding suggests that cognitive impairment on hospital admission may be a method to identify patients at increased risk for mortality using a single measure. However, cognitive impairment or mental status changes upon hospital admission may also be a marker for severe medical illness, such as sepsis (Raith et al., 2017), or delirium (Witlox et al., 2010) that may account for the observed increase in mortality. As we do not have data on the chronicity of the cognitive impairment on admission, it is difficult to ascertain whether the cognitive impairment was due to an underlying chronic dementia or whether it was an acute change due to the medical illness which prompted the hospitalization. Future research with larger populations of patients comparing admission cognitive impairment, including assessments of whether the changes in cognition are acute or chronic, with other validated predictors of mortality could be conducted to see if impairment of cognitive impairment alone would be a simple method to identify patients at risk for increased mortality after discharge.

We found that the HARP score was not associated with an increased risk for 30-day readmissions after discharge. High HARP patients are much more likely to be discharged to a skilled nursing facility after discharge (Liu et al., 2016) and potentially this in combination with the increased mortality risk at 30 days may explain why high HARP score patients had a nonsignificant lower risk of readmission within 30 days compared to the intermediate and low HARP score patients.

Other models for predicting mortality exist and have been well validated. The Geriatric Index of Comorbidity which classifies patients based on disease and severity of disease (Rozzini et al., 2002) and the Charlson Comorbidity Score which uses 19 different conditions with varied weights (Charlson et al., 1987) all use data that are not always readily available to providers, and outside of research purposes are too unwieldy or time consuming to be easily applied in routine clinical care. The main benefit of using the HARP score to predict postdischarge mortality is that it does not require calculation of a patient’s comorbidities, disease severity, hospitalization length of stay, lab data, or prior health care utilization to identify patients at a higher risk for mortality after discharge. This simple, easy to use, three item instrument that was administered by trained nursing assistants can not only identify patients who are at risk for functional decline and skilled nursing facility placement after discharge, it can also be used to help predict mortality following a hospital discharge.

The study has several limitations. First, the analysis was performed on a relatively small sample of patients who were hospitalized in one medical unit in a single academic institution with a largely rural patient population which could lead to poor generalizability. Replication of this study at other institutions with more urban or diverse patient populations or at a community based hospital would be helpful to confirm these findings. Second, self- and family-reported IADL information was used to calculate the HARP score and did not use objective or performance-based functional assessments to validate or augment this assessment. Hospitalized older adults tend to overestimate ADL function (Sager et al., 1992) which could have potentially led to the incorrect classification of patients into a lower HARP score group. Third, assessment of death and hospital readmissions was performed through chart review of the institution’s electronic health record and not through direct patient contact. This method has the potential to miss deaths and readmissions that occurred in care settings outside of the institution and result in a lower mortality or readmission rate. As a tertiary care institution, it is possible that patients were readmitted to local community hospitals which would not have been captured in our data. However, the mortality and readmission rate would not be expected to differentially affect one HARP group over the others.

The findings of this study suggest several areas of future research and possible opportunities for improvement in the care for older adults. One potential improvement initiative could entail increasing the availability and visibility of the HARP score among providers and evaluating the impact on medical decision making, patient outcomes, and future health care utilization among older patients discharged from the hospital. Furthermore, regular identification of patients at high risk for functional decline could lead to inpatient interventions that can decrease functional decline and iatrogenic complications from the hospitalization such as delirium that could potentially reduce posthospitalization mortality rates.

In the final year of life, there is evidence that patients spend more time in the hospital rather than at home (Fischer, Min, Cervantes, & Kutner, 2013). With this in mind, it is particularly disconcerting that when questioned about where they prefer to die patients overwhelmingly desire to be at home (Bruera, Russell, Sweeney, Fisch, & Palmer, 2002; Fischer et al., 2013). Thus, with better information about a patient’s prognosis following a hospitalization, we could potentially improve the delivery of appropriate evidenced-based care that is aligned with a patient’s and family’s goals of care and wishes. The innovative application of the HARP score has the potential to identify high-risk patients and creates an opportunity to improve shared decision making and end-of-life discussions and planning among providers and patients and their families.

## Funding

This work was partially funded through work supported by S. K. Liu’s participation in the Practice Change Leaders for Aging and Health Program sponsored by the Atlantic Philanthropies and the John A. Hartford Foundation. J. A. Batsis’ research reported in this publication was supported in part by the National Institute On Aging of the National Institutes of Health under Award Number K23AG051681. The content is solely the responsibility of the authors and does not necessarily represent the official views of the National Institutes of Health. S. J. Bartels receives funding from the National Institute of Mental Health (K12 HS0217695 [AHRQ], NIMH: T32 MH073553, R01 MH078052, R01 MH089811; R24 MH102794 CDC U48DP005018. This work was also supported by the Dartmouth Health Promotion and Disease Prevention Research Center (Cooperative Agreement Number U48DP005018) from the Centers for Disease Control and Prevention. The findings and conclusions in this journal article are those of the authors and do not necessarily represent the official position of the Centers for Disease Control and Prevention.

## Conflict of Interest

S. K. Liu is a consultant for The Oak Group International, Wellesley, MA. This consulting work was not related to the design, methods, analysis, or preparation of this manuscript.
